# Brain MRI Volumetry Analysis in an Indonesian Family of SCA 3 Patients: A Case-Based Study

**DOI:** 10.3389/fneur.2022.912592

**Published:** 2022-06-29

**Authors:** Siti Aminah Sobana, Fathul Huda, Robby Hermawan, Yunia Sribudiani, Tan Siauw Koan, Sofiati Dian, Paulus Anam Ong, Nushrotul Lailiyya Dahlan, Nastiti Utami, Iin Pusparini, Uni Gamayani, Norlinah Mohamed Ibrahim, Tri Hanggono Achmad

**Affiliations:** ^1^Department of Neurology, Faculty of Medicine, Dr. Hasan Sadikin Central General Hospital/Universitas Padjadjaran, Bandung, Indonesia; ^2^Research Center of Medical Genetics, Faculty of Medicine, Universitas Padjadjaran, Bandung, Indonesia; ^3^Doctoral Study Program, Faculty of Medicine, Universitas Padjadjaran, Bandung, Indonesia; ^4^Department of Biomedical Sciences, Faculty of Medicine, Universitas Padjadjaran, Bandung, Indonesia; ^5^Department of Radiology, Saint Borromeus Hospital, Bandung, Indonesia; ^6^Department of Medicine, Faculty of Medicine, Universiti Kebangsaan Malaysia Medical Center, Kuala Lumpur, Malaysia

**Keywords:** SCA3, volumetry, brain, MRI, Indonesia

## Abstract

**Introduction:**

Spinocerebellar ataxia type-3 (SCA3) is an adult-onset autosomal dominant neurodegenerative disease. It is caused by expanding of CAG repeat in ATXN3 gene that later on would affect brain structures. This brain changes could be evaluated using brain MRI volumetric. However, findings across published brain volumetric studies have been inconsistent. Here, we report MRI brain volumetric analysis in a family of SCA 3 patients, which included pre-symptomatic and symptomatic patients.

**Methodology:**

The study included affected and unaffected members from a large six-generation family of SCA 3, genetically confirmed using PolyQ/CAG repeat expansion analysis, Sanger sequencing, and PCR. Clinical evaluation was performed using Scale for the Assessment and Rating of Ataxia (SARA). Subjects' brains were scanned using 3.0-T MRI with a 3D T1 BRAVO sequence. Evaluations were performed by 2 independent neuroradiologists. An automated volumetric analysis was performed using FreeSurfer and CERES (for the cerebellum).

**Result:**

We evaluated 7 subjects from this SCA3 family, including 3 subjects with SCA3 and 4 unaffected subjects. The volumetric evaluation revealed smaller brain volumes (*p* < 0.05) in the corpus callosum, cerebellar volume of lobules I-II, lobule IV, lobule VIIB and lobule IX; and in cerebellar gray matter volume of lobule IV, and VIIIA; in the pathologic/expanded CAG repeat group (SCA3).

**Conclusion:**

Brain MRI volumetry of SCA3 subjects showed smaller brain volumes in multiple brain regions including the corpus callosum and gray matter volumes of several cerebellar lobules.

## Introduction

Spinocerebellar ataxia type-3 (SCA3) is an autosomal dominant neurodegenerative disease characterized by progressive ataxia that usually starts in early to mid-adulthood decade resulting in early wheelchair dependence and immobilization. Although it is rare in the general population with a prevalence of 1–2/100,000 it is the most common subtype of spinocerebellar ataxias (SCA) worldwide (1, 2). SCA3 patients typically present with bilateral cerebellar syndrome, characterized by gait and limb ataxia, although non-ataxia symptoms are also quite common. The non-ataxia features of SCA3 are varied and include extrapyramidal manifestations such as dystonia, parkinsonism, tremor and spasticity (3, 4).

SCA3 is caused by CAG trinucleotide repeats which exceed 46 repeats in the ATXN3 gene located at the N-terminus of chromosome 14 (1, 2, 5–8). To date, there are no curative treatment or treatments that could halt the progression of SCA3. Current treatment is still aimed at symptomatic management using pharmacological therapies; while non-pharmacological management such as various forms of rehabilitation therapies are utilized to improve balance, mobility and speech. Promising disease-modifying therapies such anti-ATXN3 aggregation compounds, ATXN3 mRNA silencing therapies, autophagy stimulation, stem cell and gene therapies are still in the pipeline and or in Phase 2 clinical trials, and are still not available (1, 2, 6, 9).

Current evaluation of patients with SCA 3 include a detailed clinical assessment, coupled with the measurement of ataxia severity using validated instruments such as the Scale for the Assessment and Rating of Ataxia (SARA) (4, 10). Genetic analysis using Sanger sequencing methods for PolyQ/CAG repeat expansion is required for confirmatory diagnosis, and the detection of the number of CAG repeats. Magnetic resonance imaging (MRI) is part of the clinical workup, although there are no specific distinguishing or diagnostic features unique for SCA3, and typically shows cerebellar atrophy (7, 11–14). An MRI with 3.0-T magnetic field, which is already available in some of the developing countries, is perhaps necessary for a reliable spatial and contrast resolution for volumetric analysis (15, 16).

Recent publications have reported variable findings in the volumetry of various anatomical brain structures in patients with SCA3 (17–24). One recent study reported that patients with SCA 3 had decreased cerebellar and cerebral volumes, and also the basal ganglia, gray matter and the thalamus (18). Given the lack of reports on MRI brain volumetry in SCA3 from the South East Asia region, especially from Indonesia, we therefore conducted this study to evaluate the MRI brain volumetry in SCA 3 patients from a large Indonesian family and compared the findings to unaffected members. We also included volumetric analysis of other brain regions than previously reported.

## Methods

This was a cross-sectional, case-based study involving a large family with SCA 3 within the Garut Regency in Indonesia.

### Ethics Statement

This study was reviewed and approved by the Research Ethics Committee of Universitas Padjadjaran no 032107017/2021. Written informed consent in accordance with the ethics committee and Declaration of Helsinki were obtained from all subjects.

### Patient Recruitment

**Figure 1:**
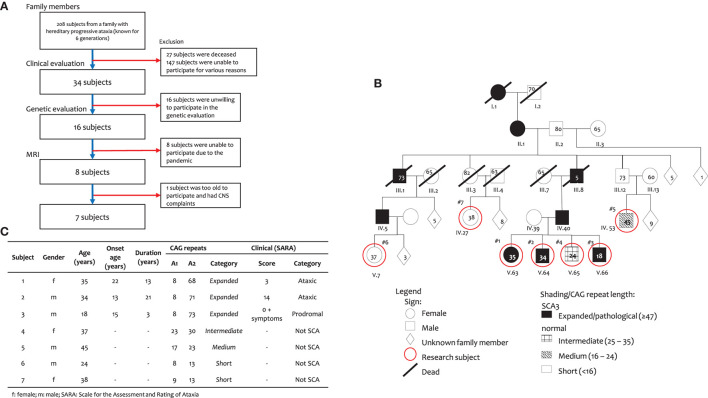
Subject of this study. **(A)** Subject inclusion, **(B)** Family pedigree, and **(C)** Subject characteristics.

Subjects of this study were from a family with progressive hereditary ataxia who were genetically confirmed with ATXN3 gene mutation. This family has 208 members; of these, 34 members had already been clinically evaluated, and 16 of these members had been genetically screened for SCA repeats. Of these 16 subjects, seven consented to take part in this volumetric study (Figures 1A,C).

### Clinical Assessment

Clinical assessment was measured using the Scale for Assessment and Rating of Ataxia (SARA). SARA consists of eight items, including tests of gait, stance, sitting, speech, finger-chase test, finger-nose test, fast alternating movements, and heel-shin test (4). According to criteria by Velazquez-Perez et al., an asymptomatic subject is a subject with SARA score of 0 and without any symptoms; those with SARA score of 0–2 and with ataxia symptoms are categorized as prodromal; while those with SARA score of 3 and above are included into ataxic group (10).

### Genetic Evaluation

Genetic evaluation was done using blood sample with polyQ/CAG repeat expansion analysis according to Sanger sequencing. According to criteria by du Montcel et al., a person has SCA3 if the CAG repeat length is *expanded* ≥ 47; otherwise, the individual is considered an unaffected subject (8).

### Brain MRI Protocols

Subjects' brain was scanned using 24 channel head coil in a 3.0-Tesla MRI scanner (Signa Pioneer; GE Healthcare, Chicago, United States). Scanning was done in the sagittal plane using isotropic 3D T1 BRAVO sequence (TR/TE 9 ms/3.5 ms, NEX 1, slice thickness 1 x 1 mm). This sequence is able to differentiate brain structure best suited for automatic identification by FreeSurfer and CERES (explained in following section). All brain MRI imaging and analysis were performed by (FH, RH) and two neuroradiology consultants (RH, TSK), who independently reviewed the images.

### Image Capture

**Figure 2:**
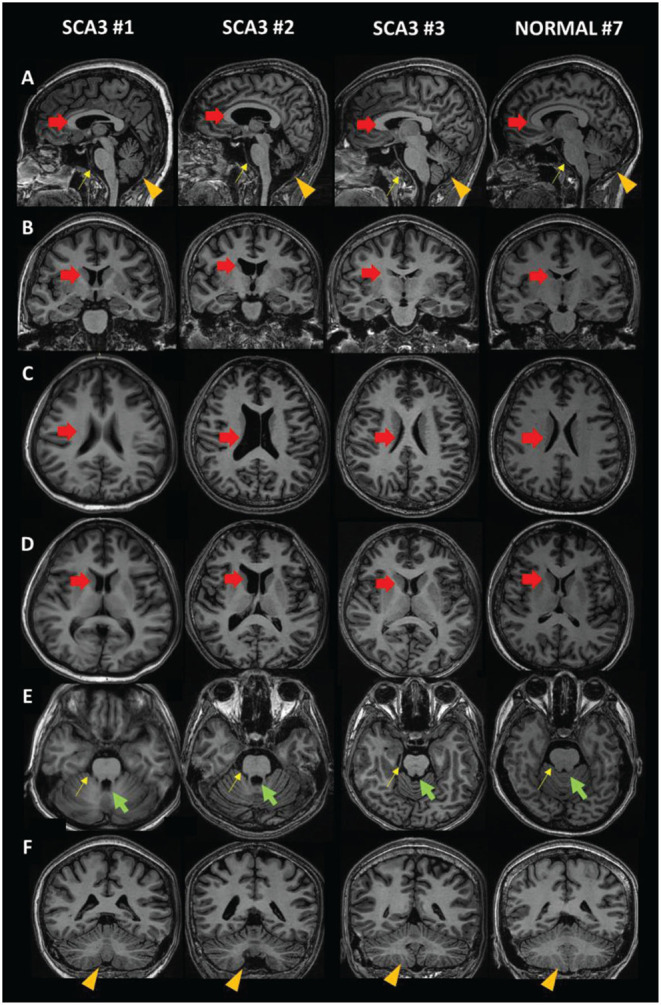
MRI images of SCA3 patients compared to a normal subject. **(A)** Sagittal view. **(B,F)** Coronal view. **(C–E)** Axial view. Red arrow: lateral ventricle (dilated in SCA3); green arrow: fourth ventricle (dilated in SCA3); yellow arrow: brainstem (size reduced in SCA3); orange arrowhead: cerebellum (size reduced in SCA3).

Captured MRI images were retrieved from DICOM images, using RadiANT ver 2020.2 (Medixant, Poznand, Polandia) (25) (Figure 2). Briefly, DICOM data were mounted to the software, then sagittal plane T1 BRAVO image sequences were loaded. Reconstruction method using multiplanar reformation or reconstruction (MPR) were done, and Talairach line was set. Image capture of certain position was acquired.

### Volumetric Calculation

Brain MRI images were analyzed automatically (except for cerebellum) using FreeSurfer ver 7.1.1, software from *Martinos Center of Biomedical Imaging*, and *Massachusetts General Hospital* / *Harvard University* (Boston, MA), https://surfer.nmr.mgh.harvard.edu/. Briefly, after installation of Freesurfer computer (Mac/Linux OS), designated T1 3D BRAVO images were loaded into the software. First, *-reconall* command was executed to get skull stripping, and brain mask feature applied to the image for volumetric evaluation. Volumetric evaluation was computed automatically which required ~8–12 h per subject. The results were marked brain region, and inspected by two independent neuroradiology consultants. If the brain marking was not satisfactory, a tweak would be done, and automatic re-evaluation would be repeated from the beginning. And if after re-evaluation, brain marking was still not satisfactory, a second brain MRI scan was performed (26, 27).

For automatic cerebellar evaluation, CERES (*Cerebellum Segmentation*) ver 1.0 was used (developed by *Universidad Politecnica de Valencia and Université de Bordeaux* [*Informatica biomedica*-IBIME, Valencia, Spanyol], https://www.volbrain.upv.es/) (28). Briefly, DICOM image were converted to NIFTI format, then loaded to VolBrain server. CERES pipeline was selected, and evaluation gray matter volume, white matter volume, and cortical thickness of each cerebellar lobule was done automatically.

### Volumetric Evaluation

Volumetric data calculated by FreeSurfer and CERES were processed and compared with the percentage intracranial volume that was provided by the volumetric analysis. Brain volumetry of the anatomical structures in this study was calculated as ratio of the individual subject's intracranial volume (ICV). The volume ratio is used since there is no standard or normal range for the volume of the various brain structures yet. In addition, the shape of the head and the volume of the human brain varies. Therefore, an objective standard is needed in the volumetric measurement of the brain. This can be achieved by measuring the ratio of volume relative to ICV. Since we use this ICV as the denominator in the volume calculation, so we name it percentage to ICV (%ICV), to be more precise.

### Heat Map

In order to make the assessment easier, a heat-map color grading pattern was employed (Tables 1A,B). Briefly, the cells in the table are colored by conditional formatting using color scale feature, with green for the largest number gradating to yellow and red for the smallest number.

**Table 1A:** Brain MRI volumetry. **(A)** Cerebrum and brainstem.

**Group**	**Subject** **No**	**Thalamus**	**Basal** **Ganglia**	**Ventricle**	**Brainstem**	**Hippo-campus**	**Amygdala**	**nucleus** **Accumbens**	**Diencephalon**	**Corpus** **Callosum**	**Cortical** **Volume**	**White** **Matter**	**Subcortical** **Gray Matter**	**Total Gray** **Matter Volume**
SCA3	1	1.05%	1.42%	1.51%	1.51%	0.61%	0.19%	0.09%	0.54%	0.20%	32.93%	29.57%	4.02%	44.78%
	2	0.90%	1.26%	2.33%	1.26%	0.55%	0.21%	0.07%	0.48%	0.20%	29.90%	30.25%	3.59%	40.17%
	3	1.12%	1.58%	1.03%	1.46%	0.56%	0.22%	0.07%	0.57%	0.23%	36.34%	31.68%	4.22%	47.92%
Normal	4	1.06%	1.63%	1.32%	1.63%	0.61%	0.24%	0.08%	0.57%	0.24%	31.82%	31.67%	4.32%	44.13%
	5	0.99%	1.38%	0.97%	1.53%	0.55%	0.20%	0.07%	0.54%	0.23%	30.95%	32.41%	3.84%	42.46%
	6	0.99%	1.49%	1.33%	1.49%	0.50%	0.22%	0.08%	0.57%	0.24%	31.84%	30.94%	3.98%	42.73%
	7	1.07%	1.44%	0.88%	1.50%	0.51%	0.23%	0.09%	0.58%	0.25%	29.74%	31.99%	4.04%	41.33%
	*p-value*	0.571	0.314	0.114	0.114	0.314	0.114	0.429	0.114	0.029*	0.200	0.114	0.429	0.314

*p < 0.05.

**Table 1B:** Cerebellum.

**Group**	**Subject No**	**Lobule I-II**	**Lobule III**	**Lobule IV**	**Lobule V**	**Lobule VI**	**Lobule Crus I**	**Lobule Crus II**	**Lobule VIIB**	**Lobule VIIIA**	**Lobule VIIIB**	**Lobule IX**	**Lobule X**	**Total**
**Volume (%ICV)**															
SCA3	1	0.01%	0.08%	0.32%	0.59%	1.30%	1.72%	1.39%	0.71%	0.80%	0.52%	0.48%	0.08%	8.99%
	2	0.01%	0.10%	0.26%	0.50%	1.11%	1.55%	1.13%	0.59%	0.79%	0.51%	0.44%	0.06%	7.98%
	3	0.01%	0.08%	0.33%	0.56%	1.16%	1.62%	1.07%	0.57%	0.90%	0.55%	0.45%	0.07%	8.42%
Normal	4	0.01%	0.09%	0.34%	0.58%	1.35%	2.03%	1.16%	0.78%	0.89%	0.60%	0.53%	0.09%	9.73%
	5	0.01%	0.09%	0.34%	0.55%	1.27%	1.80%	1.11%	0.58%	0.86%	0.62%	0.66%	0.09%	9.09%
	6	0.01%	0.08%	0.35%	0.58%	1.15%	1.45%	1.18%	0.62%	0.82%	0.56%	0.48%	0.07%	8.50%
	7	0.01%	0.10%	0.35%	0.59%	1.39%	2.03%	1.14%	0.66%	0.82%	0.68%	0.64%	0.07%	9.72%
	*p-value*	0.029*	0.2	0.029*	0.314	0.2	0.2	0.429	0.314	0.314	0.029*	0.029*	0.114	0.057
**Gray Matter Volume (%ICV)**															
SCA3	1	0.00%	0.06%	0.29%	0.54%	1.18%	1.50%	1.21%	0.63%	0.72%	0.45%	0.41%	0.07%	7.06%
	2	0.00%	0.08%	0.24%	0.44%	1.01%	1.36%	0.98%	0.53%	0.70%	0.45%	0.36%	0.05%	6.21%
	3	0.00%	0.07%	0.28%	0.46%	1.10%	1.58%	0.90%	0.63%	0.72%	0.48%	0.40%	0.07%	6.70%
Normal	4	0.00%	0.09%	0.35%	0.57%	1.33%	1.83%	1.14%	0.60%	0.89%	0.62%	0.63%	0.09%	8.15%
	5	0.00%	0.06%	0.29%	0.48%	1.04%	1.37%	0.91%	0.50%	0.78%	0.47%	0.36%	0.06%	6.34%
	6	0.00%	0.06%	0.32%	0.51%	1.05%	1.29%	1.05%	0.56%	0.73%	0.49%	0.41%	0.06%	6.53%
	7	0.01%	0.08%	0.30%	0.53%	1.26%	1.78%	0.98%	0.58%	0.72%	0.58%	0.53%	0.07%	7.44%
	*p-value*	0.314	0.571	0.029*	0.2	0.314	0.429	0.429	0.2	0.029*	0.057	0.2	0.571	0.314
**Cortical Thickness (mm)**															
SCA3	1	2.03	3.48	5.12	5.12	5.12	4.97	4.97	5.00	4.96	5.04	4.64	3.63	4.97
	2	3.56	3.96	5.21	5.11	5.12	5.11	5.02	4.94	4.87	5.04	4.43	3.47	4.98
	3	1.95	3.66	5.18	5.14	5.25	5.04	5.02	5.18	5.03	5.01	4.74	3.23	5.04
Normal	4	2.38	3.70	5.22	5.15	5.21	5.31	5.23	5.12	5.00	5.18	4.94	3.71	5.14
	5	2.28	3.55	5.13	5.01	5.15	5.02	4.84	5.03	4.87	5.04	4.52	3.22	4.94
	6	1.16	3.18	5.22	4.91	5.19	5.17	5.18	5.15	5.04	4.98	4.86	3.51	5.07
	7	2.52	3.30	5.07	5.17	5.17	5.10	4.83	4.96	4.93	4.97	4.54	3.63	4.97
	*p-value*	0.571	0.2	0.371	0.571	0.257	0.2	0.543	0.429	0.486	0.457	0.314	0.371	0.457

*Volume is presented as a percentage of intracranial volume; cortical thickness is presented in mm*.

*A heatmap color scale is used. *p < 0.05.
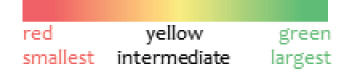
*

### Statistical Analysis

Since the sample size is small, the non-parametric Mann-Whitney U test was used to compare two groups in terms of quantitative variables. Data were analyzed using SPSS ver 25.0 for Windows (IBM Corp. IBM SPSS Statistics fo Windows, Version 25.0 Armonk, NY: IBM Corp). Mann-Whitney U test was employed for non-parametric. *P* < 0.05 is considered statistically significance.

## Result

Our subjects came from a large six-generation family with 208 members with a history of progressive cerebellar syndrome, genetically confirmed as SCA3. Clinically affected family members were one person from the first generation; two members from the second generation, 10 members from the third generation, 54 people in the fourth generation, 89 people in the fifth generation and 11 people, thus far, in the sixth generation. The relevant pedigree in shown in (Figures 1A,B).

Out of those 208 members, only seven were willing to take part in this volumetric study (Figure 1A). Subjects 1–3 are SCA3 patients and siblings with Subject 4. They are descendants from a father (IV.40), paternal grandfather (III.8), great grandmother (II.1) and great-great grandmother (I.1), who had ataxia complaints. Subjects 5–7 are unaffected. Subject 5 had parents and grandparents with no ataxia complaints. Subject 6 is a descendant of a father (IV.5), paternal grandfather (III.1), great grandmother (II.1) and great-great grandmother (I.1), who had progressive ataxia complaints. The parents of Subject 7 had no ataxia complaint, despite a great grandmother (II.1) and great-great grandmother (I.1), who had ataxia complaints.

Genetic evaluation showed that subjects 1–3 were confirmed as SCA3 subjects (Figure 1C) as they had heterogeneous alleles, with one of the alleles having 68, 71 and 73 expanded CAG repeats, respectively. The other allele had 8 repeats in all three subjects. Subject 4 had an intermediate number of repeats with 23 and 30 CAG repeats. Subject 5 had medium number of CAG repeats (17 and 23), while Subjects 6 and 7 are included as short CAG repeats (13).

From a clinical point of view, according to criteria by Velazquez-Perez et al. (10), Subjects 1 and 2 are considered to have genetically confirmed SCA 3, while Subject 3 is considered prodromal. All other subjects were considered as unaffected. The clinical details of SCA 3 subjects and controls are shown in (Figure 1C).

The ages of subjects 1, 2 and 3 were 35, 34 and 18 years old, respectively and the onset of ataxia symptoms were at 22, 13 and 15 years of age, with disease duration of 13, 21, and 3 years, respectively. The clinical features of all three subjects are summarized in (Figure 1C). Subject 1, a female had gait ataxia and balance difficulties and muscle cramps, with mild cognitive impairment (MoCA-Ina: 22; MMSE: 24) and a SARA score of 3 (gait: 1; standing: 1; and heel-shin coordination: 1). Subject 2, male, is considered ataxic, complaining of walking and postural problems with a severe talking and mild/moderate coordination problems, with mild cognitive impairment (MoCA-Ina: 23) and a SARA score of 14 (gait: 4; standing: 2; talk: 3; finger chase: 1; nose-finger: 1; hand alternating movement: 1; and heel-shin: 2). Subject 3, male, is considered prodromal and, despite a normal motor condition, has had cognitive complaints from 15 years old (MoCA-Ina 22; MMSE: 21), and a SARA score of 0, plus numbness on his skin. All other subjects (4–7) had <47 CAG repeats, so they were considered unaffected subjects.

Qualitatively, we compared MRI brain images from SCA3 subjects (Subjects 1–3) and a unaffected subject (Subject 7) (Figure 2). Dilation of the lateral ventricle (red arrow) and fourth ventricle (green arrow) was observed in Subjects 1 and 2. We also observed smaller brainstem (yellow arrow) and cerebellar (orange arrowhead) volumes. In contrast, the quantitative MRI brain image of Subject 3 is presumably equal to that of a unaffected subject. This might be due to the duration of disease and young age.

Detailed brain volumetric results of subjects grouped by CAG repeats and clinical data can be found in (Tables 1A,B) Statistical analysis showed a statistical significance difference between SCA3 and unaffected subjects in volume of corpus callosum, and cerebellum (lobules I-II, lobule IV, lobule VIIB and lobule IX) and also in volume in gray matter of cerebellum (lobule IV, and VIIIA) but not in other measurements.

## Discussion

SCA3 is a neurological disease which begins as early as the 2nd decade, characterized by progressive cerebellar syndrome and cerebellar atrophy. Brain atrophy occurs as a result of a toxic gain-of-function, mitochondrial bioenergetic problems, ion channel disturbance, RNA toxicity, repeat-associated non-AUG (RAN) translation, transcription problems, DNA damage, protein transport disturbance and altered ubiquitination processes in the brain. This leads to axonal damage and neuronal and glial death (2, 8). Cortical thinning in SCA3 patients has been associated with the death of Purkinje cells as the sole processing neuron in the cerebellum and damage to cortical glia (Bergmann's glia, stellate cells and basket cells), which contributes to progressive incoordination and ataxia (29, 30).

In this study, we were able to compare brain volumetric findings among members from the same family, who were clinically affected and had genetic confirmation of SCA 3 with unaffected subjects who did not carry SCA 3 genetic mutation. Expectedly, the cerebellar volume of the SCA3 subjects was lower than that of the unaffected subjects. There were however variations in the lobules affected among the three subjects affected by SCA 3. Motor and cognitive function are currently known to be controlled not only by the cerebrum but also by the cerebellum. According to the double motor and triple non-motor concept of cerebellar function, the cerebellar lobules are divided into two *motor* areas: (1) lobules I-VI and (2) lobule VIII, while the three non-motor areas are (1) lobule VI/crus I, (2) crus II/lobule VIIB, and (3) lobules IX/X (31–33).

In Subject 1, with an SCA3 duration of 13 years, there were lower brain volumes in almost all motor lobules with partial atrophy in the cognition lobules. Atrophy in the motor regions was in accordance with the ataxia complaints; however, there were some discrepancies in the lobules that command cognition and the subject's cognitive condition. In Subject 2, the volumetric findings corresponded to the motor and cognitive conditions. In Subject 3, the cognitive findings were in accordance with the volumetric findings, although the motoric findings were not in accordance with the volumetric findings (Table 1C).

**Table 1C:** Cerebellar atrophy SCA3 subjects.

**Subject No**.	**1**	**2**	**3**	**Function**
Lobules I-II	v	v	v	Motor
Lobule III	v	-	v	Motor
Lobule Iv	v	v	v	Motor
Lobule v	-	v	o	Motor
Lobule VI	-	v	o	Motor/cognitive
Crus I	-	o	o	Cognitive
Crus II	-	o	v	Cognitive
Lobule VIIB	-	o	v	Cognitive
Lobule VIIIA	v	v	-	Motor
Lobule VIIIB	v	v	v	Motor
Lobule IX	v	v	v	Cognitive
Lobule X	-	v	v	Cognitive

*v = smaller volume than in normal subjects*.

*o = smaller volume than all but 1 normal subject*.

*- = approximately the same volume as in normal subjects*.

Based on recent publications, there have been different findings on volumetric differences in various brain anatomical structure of patients with SCA3 (17–24) (Table 2). Researchers from many regions reported their findings, however there were no report found on ASEAN populations. Wan et al. reviewed 12 publications with a total of 252 subjects and found atrophy in the cerebellum, brain stem, cerebrum, limbic system, basal ganglia and thalamus (19). Klaes et al. reviewed 4 volumetric studies with 246 subjects and found brain atrophy in the cerebellum and brainstem (20). Arruda et al. studied 19 SCA3 subjects in a Brazilian population and showed atrophy in the brainstem and cerebellum (18). Rezende et al. studied 43 SCA3 subjects in a Brazilian population and found vast atrophy in cortical and subcortical regions, including the cerebellum, thalamus, putamen, globus pallidum, diencephalon and brainstem (21). Guo et al. studied 47 SCA3 subjects in a Chinese population and showed progressive atrophy in cerebellar gray matter and several cerebrum regions (22). Faber et al. gathered 210 SCA3 subjects from 8 countries (Brazil, China, France, Germany, Netherland, Spain, UK, and US) and found atrophy in cerebellum, brainstem, thalamus, basal ganglia with dilation of ventricle (23). Hernandez-Castillo et al. studied 17 SCA3 patients from Mexican population and found atrophy in cerebellum, pons and left lingual gyrus (24). Overall there is an agreement that there is decreased cerebellar and cerebral volumes with various other affected regions and with varying degree. Hence, we evaluated MRI brain volumetry with more region than existing publication in SCA3 patients, moreover there are no study examining brain MRI of ASEAN and Indonesian origin. A more thorough evaluation with tractography and an analysis with all the related motor and cognitive brain regions is necessary to clarify the discrepancies observed.

**Table 2:** Findings in this study compared to other studies.

	**This study**	**Wan et al**.	**Klaes et al**.	**Arruda et al**.	**de Rezende et al**.	**Guo et al**.	**Faber et al**.	**Hernandez- Castillo et al**.
Study	Indonesian population	Review of 12 studies	Review of 4 studies	Brazilian population	Brazilian population	Chinese population	Brazil, China, France, Germany, Netherland, Spain, UK, and US	Mexican Population
Number of subjects	3 SCA3 patients, 4 normal subjects	252 SCA3 patients, 250 normal subjects	246 SCA3 patients, 246 normal subjects	19 SCA3 patients, 19 normal subjects	49 SCA3 patients, 49 normal subjects	47 SCA3 patients, 49 normal subjects	210 SCA3 patients, 63 healthy subject	17 SCA3 patients, 17 healthy subject
Journal/year	-	*FNeu*: 2020	*Am J Neurorad*: 2016	*Cerebellum*: 2020	*Eur J Neu*: 2014	*Neurology*: 2020	Mov Dis: 2021	Cerebellum Ataxias: 2017
Brain region	**Cerebellum – Gray matter (lobule V, VIIIA)** ↓*	Cerebellum ↓ (100%)	Cerebellum – hemisphere ↓ (100%) Dentate nucleus ↓(25%)	Cerebellum – cortex ↓	Cerebellum – cortex ↓	Cerebellum – cortex ↓	Cerebellum – volume ↓	Crus II, vermis IX, lobule I-IV – gray ↓
	**Cerebellum (lobules I-II, IV, VIIIB, IX)** ↓*		Cerebellum – vermis ↓(75%)	Cerebellum – white matter↓	Cerebellum – white matter↓			
	Cerebellum – cortical thickness – ns		Cerebellum – peduncle ↓(50%)					
	Brainstem – ns	Brainstem ↓ (75%)	Brainstem ↓(100%) Midbrain ↓(50%) Pons ↓(75%) Medulla oblongata ↓(50%)	Brainstem ↓	Brainstem ↓		Midbrain ↓ Pons ↓ Medulla oblongata ↓	Pons ↓
	Ventricle – ns						Ventricle ↑	
	Cerebrum – white matter– ns	Cerebrum ↓ (67%)				Cerebrum – cortex ↓		Left lingual gyrus ↓
	Thalamus – ns	Thalamus ↓ (25%)		Thalamus – ns	Thalamus ↓		Thalamus ↓	
	Basal ganglia – ns	Basal ganglia ↓ (25%)		Pallidum – ns	Caudate nucleus, putamen, pallidum ↓		Caudate nucleus, pallidum ↓	
	Amygdala – ns	Limbic regions↓ (42%)						
	Nucleus accumbens – ns							
	Diencephalon – ns				Diencephalon ↓			
	Corpus callosum ↓^*^							
	Subcortical gray matter – ns			Subcortical & total gray matter				

↓: Volume decrease; *: p < 0.05; ns, not significant; (%), percentage of patients showing the indicated change.

Our patients had onset of disease in the second and third decades, which is relatively early compared to the average published mean age of onset which is in the fifth decade but still fell within the age range of second to sixth decade. Subject 3 was the youngest, with the shortest duration of disease, and therefore had unclear amount of brain atrophy, as age and duration of disease affect the degree of brain atrophy (8, 34–36).

## Limitations of Study

Being case-based and a pilot study, our study is limited by a small sample size, which was affected further by the COVID-19 pandemic during the study period. The COVID-19 pandemic hindered the ability of family members to participate and be examined, due to transportation limitations and isolation protocols from the regional government, in the efforts to prevent the spread of COVID-19 pandemic. A study with larger sample size would be necessary for a more generalizable conclusion.

Despite the limitations, we believe our study findings could contribute to the existing literature on brain volumetry and clinical details of SCA 3 patients, in the South East Asia region, given the paucity of data in this region. To our knowledge, this is the first volumetric evaluation of SCA3 patients conducted in Indonesia. Additionally, as we had also included more brain regions in our evaluation as compared to previous studies, we measured the thalamus, basal ganglia, ventricles, brainstem, amygdala, nucleus accumbens, diencephalon, corpus callosum, white matter, and subcortical gray matter, in addition to the measurement of cerebellar volume, cerebellar gray matter volume and cerebellar cortical thickness. Statistically significant differences were found in volume of corpus callosum, and cerebellum (lobules I-II, lobule IV, lobule VIIB and lobule IX) and also in volume in gray matter of cerebellum (lobule IV, and VIIIA) but not in other measurements.

In conclusion, our findings confirm previous findings that various brain regions are atrophied in SCA 3, and perhaps may involve other parts of the brain not previously reported. However, our findings remain preliminary and need to be verified further in larger studies. Brain MRI volumetric analysis could thus be an additional tool for non-invasive evaluation of patients with degenerative cerebellar ataxia in addition to the currently utilized clinical scales (20, 37).

## Data Availability Statement

The original contributions presented in the study are included in the article material, further inquiries can be directed to the corresponding authors.

## Ethics Statement

The studies involving human participants were reviewed and approved by Universitas Padjadjaran, Research Ethic Committee. The patients/participants provided their written informed consent to participate in this study.

## Author Contributions

SS: study design, conceptualization, data collection, data analysis, manuscript revision, and funding. FH: study design, conceptualization, data collection, data analysis, manuscript drafting, and manuscript revision. TK: study design, data collection, and data analysis. RH: study design, data collection, data analysis, and manuscript drafting. YS: study design, conceptualization, data analysis, manuscript drafting, manuscript revision, and funding. SD, PO, and ND: data analysis and manuscript revision. IP: study design, data collection, and manuscript revision. UG: study design, data analysis, and manuscript revision. NM: conceptualization and manuscript revision. TA: study design, conceptualization, manuscript revision, and funding. All authors contributed to the article and approved the submitted version.

## Funding

This research was funded by National Research and Innovation Agency–Ministry of Education, Culture, Research and Technology of Indonesia, via Universitas Padjadjaran no 1207/UN6.3.1/PT.00/2021 for Tri Hanggono Achmad.

## Conflict of Interest

The authors declare that the research was conducted in the absence of any commercial or financial relationships that could be construed as a potential conflict of interest.

## Publisher's Note

All claims expressed in this article are solely those of the authors and do not necessarily represent those of their affiliated organizations, or those of the publisher, the editors and the reviewers. Any product that may be evaluated in this article, or claim that may be made by its manufacturer, is not guaranteed or endorsed by the publisher.

## Abbreviations

ATXN3: ataxin3 (could refer to gene or protein)
CAG: trinucleotide repeat of CAG
CERES: Cerebellum Segmentation
MMSE: Mini-Mental State Examination
MoCA-Ina, Montreal Cognitive Assessment: Indonesian version
MRI: magnetic resonance imaging
PolyQ: polyglutamine
SARA: Scale for the Assessment and Rating of Ataxia
SCA: spinocerebellar ataxia.
